# Bypassing D-dimer Testing in Suspected High-Risk Pulmonary Embolism in the Emergency Department: A Case Report

**DOI:** 10.7759/cureus.103190

**Published:** 2026-02-08

**Authors:** Stanislaw Szymkiewicz, Michal Wróbel

**Affiliations:** 1 Department of Urology, Janusz Korczak Provincial Specialist Hospital, Slupsk, POL; 2 Surgery, Wielospecjalistyczny Szpital Miejski im. Józefa Strusia, Poznań, POL

**Keywords:** computed tomography, d-dimer, emergency department, mechanical thrombectomy, pulmonary angiography, pulmonary embolism, risk stratification

## Abstract

Pulmonary embolism (PE) remains a leading cause of sudden hemodynamic deterioration and death in emergency department patients. Although diagnostic algorithms frequently incorporate D-dimer testing, in patients with high clinical probability or hemodynamic instability, definitive imaging should not be delayed. We report a case of a 74-year-old man presenting with syncope, hypoxemia, and hypotension, in whom immediate computed tomography pulmonary angiography (CTPA) was performed without prior D-dimer testing due to strong clinical suspicion of high-risk PE. Imaging revealed extensive bilateral pulmonary emboli with radiological signs of right ventricular strain, which were subsequently supported by point-of-care echocardiography. Due to recent head trauma, systemic thrombolysis was contraindicated, and the patient was referred for urgent mechanical thrombectomy. This case highlights the importance of clinical judgment and early imaging in high-risk PE and emphasizes that laboratory testing should not delay life-saving diagnostic and therapeutic decisions in unstable patients.

## Introduction

Pulmonary embolism (PE) is a potentially fatal condition that frequently presents with nonspecific symptoms, making diagnosis challenging in the emergency department (ED) [1,2]. Diagnostic strategies commonly combine clinical probability assessment with D-dimer testing and imaging, allowing exclusion of PE in patients with low or intermediate pretest probability [3-5]. However, in patients with high clinical suspicion or hemodynamic instability, D-dimer testing has limited diagnostic value and may delay definitive diagnosis and treatment [5,6].

Historically, the term “massive pulmonary embolism” has been used to describe PE associated with hemodynamic instability; in the current European Society of Cardiology (ESC) guidelines, this entity is classified as high-risk PE. Current ESC and emergency medicine guidelines recommend immediate imaging, most commonly computed tomography pulmonary angiography (CTPA), or bedside echocardiography as an adjunctive tool, when PE is suspected in unstable patients, without delaying management for laboratory confirmation [5]. Rapid diagnosis and early reperfusion therapy are critical determinants of outcome in high-risk PE [2]. This case illustrates appropriate deviation from stepwise diagnostic algorithms in favor of immediate imaging based on clinical presentation.

## Case presentation

A 74-year-old man with a medical history of hypertension, benign prostatic hyperplasia, and previous venous thromboembolism presented to the ED after several syncopal episodes accompanied by progressive dyspnea over one week, which had worsened to dyspnea at rest on the day of admission. During ambulance transport, the patient developed transient apnea without cardiac arrest and required brief bag-valve-mask ventilation, followed by spontaneous recovery of breathing and consciousness.

On arrival, the patient was hypotensive (initial blood pressure 80/40 mmHg, improving to 99/55 mmHg), tachycardic (heart rate 110-120 beats per minute), and hypoxemic with an oxygen saturation of 85% on room air, improving with high-flow oxygen via a non-rebreather mask. Physical examination revealed signs of shock without focal neurological deficits, although superficial head trauma was noted following syncope.

**Figure 1:**
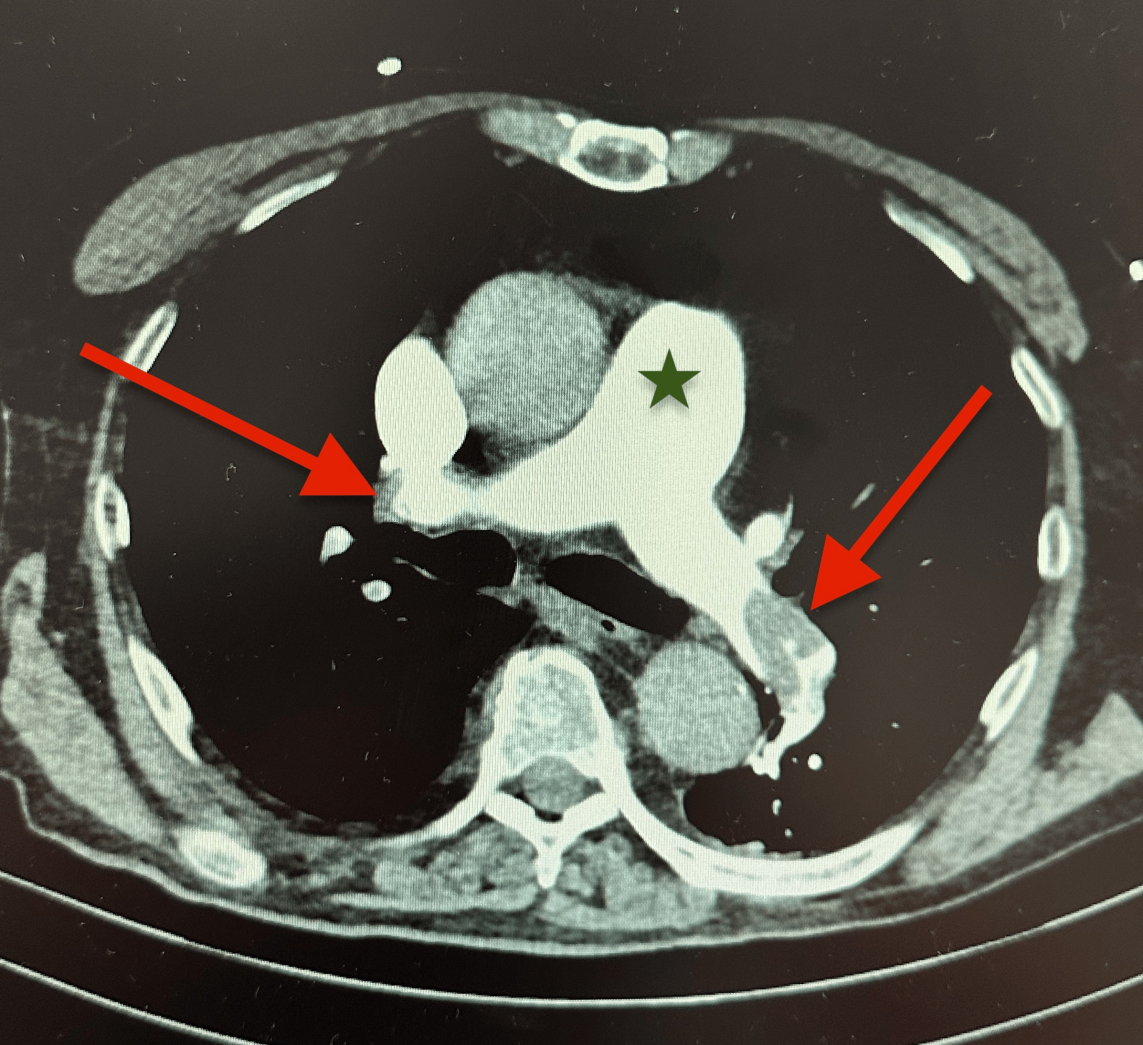
Computed tomography pulmonary angiography demonstrating acute pulmonary embolism. Axial computed tomography pulmonary angiography (CTPA) showing extensive intraluminal filling defects within both main pulmonary arteries (arrows) and dilation of the main pulmonary artery, consistent with acute pressure overload in high-risk pulmonary embolism.

**Figure 2:**
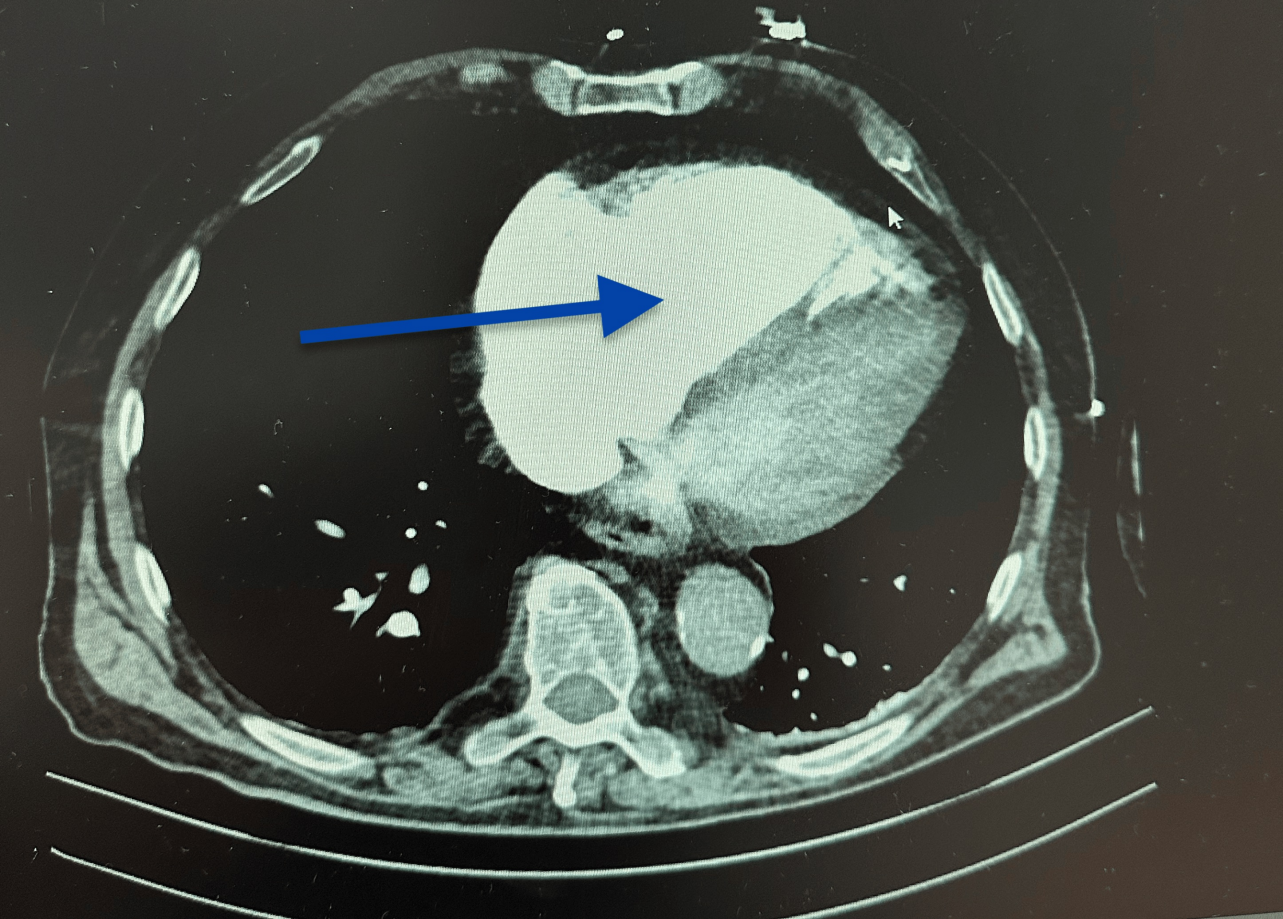
Right ventricular enlargement on computed tomography pulmonary angiography (CTPA). Right ventricular enlargement on computed tomography pulmonary angiography. Axial computed tomography pulmonary angiography (CTPA) demonstrating marked enlargement of the right ventricle (arrow) relative to the left ventricle, consistent with right ventricular strain and adverse prognostic features in acute high-risk pulmonary embolism.

The combination of syncope, sustained hypotension, hypoxemia, and a history of venous thromboembolism raised a high clinical suspicion of high-risk PE. Other immediately life-threatening conditions, including acute coronary syndrome, aortic dissection, intracranial hemorrhage, and primary arrhythmia, were considered and rapidly excluded based on clinical assessment and initial investigations. Given the patient’s hemodynamic instability, immediate CTPA was performed without prior D-dimer testing, in accordance with guideline recommendations for suspected high-risk PE [5]. Imaging demonstrated extensive bilateral pulmonary emboli involving the main pulmonary arteries and lobar branches, with enlargement of the pulmonary trunk and radiological signs of right ventricular strain (Figures 1-2).

Following CTPA confirmation of PE, point-of-care echocardiography was performed and revealed right ventricular dilation, reduced tricuspid annular plane systolic excursion (TAPSE 14 mm), shortened pulmonary acceleration time, and a positive McConnell sign, while left ventricular systolic function was preserved. These findings were consistent with right ventricular dysfunction and adverse prognostic features in high-risk PE [2].

Laboratory tests, including D-dimer, NT-proBNP, and cardiac troponin, were obtained after CTPA confirmation and demonstrated marked elevation; however, these results did not influence diagnostic or therapeutic decision-making.

Computed tomography of the head showed no intracranial hemorrhage but confirmed recent superficial head trauma, representing a contraindication to systemic thrombolysis. Given the diagnosis of high-risk PE and the contraindication to thrombolytic therapy, the patient was referred for urgent mechanical thrombectomy and transferred to a tertiary cardiac center under continuous hemodynamic monitoring and intravenous anticoagulation.

## Discussion

Diagnostic pathways and the role of D-dimer

Diagnostic algorithms for suspected PE are designed to reduce unnecessary imaging in low-risk patients. In hemodynamically stable patients with low or intermediate pretest probability, D-dimer testing can safely exclude PE when negative [3-5]. However, in patients with high clinical probability or hemodynamic instability, D-dimer testing does not meaningfully influence management and may delay definitive imaging and treatment [4,5].

ESC guidelines recommend that patients with suspected high-risk PE undergo immediate imaging, most commonly CTPA, or bedside echocardiography as an adjunctive tool, without prior D-dimer testing [5]. In unstable patients, rapid diagnosis enables prompt initiation of reperfusion therapy, which is critical for survival. Overreliance on laboratory-based algorithms in such scenarios may lead to harmful delays.

Risk stratification: high versus intermediate/low risk

Risk stratification is central to PE management. According to the ESC guidelines, high-risk PE is defined by sustained hypotension, shock, or cardiac arrest and is associated with high early mortality [2,5]. Intermediate-risk PE includes patients with right ventricular dysfunction or elevated cardiac biomarkers without hemodynamic instability, while low-risk patients lack both features [2].

In the present case, the patient fulfilled ESC criteria for high-risk PE based on sustained hypotension, syncope, hypoxemia, and imaging evidence of right ventricular dysfunction, warranting immediate diagnostic and therapeutic escalation.

Diagnostic yield of CTPA in high pretest probability

The diagnostic yield of CTPA varies substantially across pretest probability groups. In patients with high clinical probability of PE, the rate of negative CTPA findings is low, reflecting a high diagnostic yield, whereas negative CTPA rates are significantly higher in low- and intermediate-risk populations. This distinction supports the use of immediate imaging in unstable, high-risk patients and further underscores the limited utility of D-dimer testing in this context.

Treatment considerations

Systemic anticoagulation remains the cornerstone of treatment for most patients with acute PE and should be initiated promptly unless contraindicated [2]. Unfractionated heparin is often preferred in unstable patients due to its rapid onset and ease of reversal if invasive procedures are required.

Systemic thrombolysis is recommended for patients with high-risk PE and hemodynamic compromise, as it can rapidly reduce thrombus burden and improve right ventricular function [2,5]. However, it carries a significant risk of major bleeding, particularly intracranial hemorrhage, and is contraindicated in patients with recent trauma or bleeding.

For patients with contraindications to systemic thrombolysis or in cases of failed thrombolysis, catheter-directed therapies and mechanical thrombectomy represent important alternative reperfusion strategies. These approaches aim to rapidly reduce clot burden while potentially lowering systemic bleeding risk. Increasing evidence supports their safety and efficacy in selected patients with high-risk PE, particularly when managed within pulmonary embolism response teams (PERT) [7,8].

Surgical embolectomy remains an option for selected patients when thrombolysis and catheter-based interventions are not feasible or have failed, especially in cases of massive central emboli with cardiovascular collapse [5].

Radiological markers and emerging risk stratification tools

Beyond confirming the diagnosis, CTPA increasingly provides valuable information for risk stratification in acute PE. Radiological parameters such as right-to-left ventricular diameter or volume ratios, pulmonary artery diameter, and pulmonary artery-to-aorta ratios have been shown to correlate with right ventricular dysfunction and adverse outcomes. These imaging-based markers may complement or, in selected settings, partially substitute echocardiography, particularly when immediate bedside echocardiography is not available. The present case aligns with this evolving paradigm by illustrating how radiological signs of right ventricular strain can support early risk assessment in high-risk PE.

Clinical implications

This case demonstrates that in patients presenting with clinical features strongly suggestive of high-risk PE, diagnostic and therapeutic decisions should be driven by clinical urgency rather than rigid adherence to stepwise algorithms. Immediate imaging without prior D-dimer testing enabled rapid diagnosis and timely referral for reperfusion therapy, consistent with the current ESC guidelines and best practices in emergency medicine [2,5].

## Conclusions

In patients with suspected high-risk PE and hemodynamic instability, diagnostic and therapeutic decisions should be based on clinical presentation and immediate imaging rather than routine laboratory screening tests. D-dimer testing should not delay definitive diagnosis or reperfusion therapy in unstable patients. Prompt recognition and expedited, guideline-based management are essential to improving outcomes in high-risk PE.
